# Association between diffuse idiopathic skeletal hyperostosis and thoracic kyphosis in patients with cervical myelopathy: a retrospective observational study

**DOI:** 10.1186/s12891-021-04851-z

**Published:** 2021-11-18

**Authors:** Motoyoshi Takayuki, Hirai Takashi, Yoshii Toshitaka, Inose Hiroyuki, Matsukura Yu, Egawa Satoru, Kobayashi Yutaka, Utagawa Kurando, Hashimoto Jun, Kawabata Atsuyuki, Takahashi Takuya, Tanaka Tomoyuki, Okawa Atsushi

**Affiliations:** Department of Orthopedic Surgery, Tokyo Medical and Dental University, 1-5-45 Yushima, Bunkyo-ku, Tokyo, 113-8519 Japan

**Keywords:** Diffuse idiopathic skeletal hyperostosis, Sagittal alignment, Ossification of the posterior longitudinal ligament, Spondylosis

## Abstract

**Background:**

Diffuse idiopathic skeletal hyperostosis (DISH) is a structural abnormality of the thoracic spine that is known to impair posture. However, the relationship between DISH and sagittal balance in the whole spine is unclear. The aims of this study were to investigate the prevalence of DISH in patients with cervical myelopathy caused by cervical ossification of the posterior longitudinal ligament (OPLL) or cervical spondylosis and to compare sagittal alignment of the spine between patients with and without DISH.

**Methods:**

A total of 103 consecutive patients with a diagnosis of cervical myelopathy due to cervical OPLL or spondylosis were retrospectively enrolled in this single-center study. DISH was defined as an ossified lesion that was seen to be completely bridging at least four contiguous adjacent vertebral bodies in the thoracic spine on computed tomography scans. Cervical and spinopelvic sagittal parameters were measured in whole spine radiographs.

**Results:**

The study population included 28 cases with DISH [DISH (+) group] and 75 without DISH [DISH (−) group]. OPLL was more prevalent in the DISH (+) group than in the DISH (−) group; however, there were no significant differences in other clinical findings. Propensity score matching produced 26 pairs. C7 slope, C2-7 sagittal vertical axis (C-SVA), whole thoracic kyphotic angles, upper thoracic kyphosis, and T5-T12 thoracic kyphosis values were significant higher in the DISH (+) group than in the DISH (−) group. There was no significant between-group difference in the other sagittal spinopelvic parameters.

**Conclusions:**

This study is the first to compare sagittal alignment in patients with cervical myelopathy according to whether or not they have DISH. Patients with DISH are more likely to have excessive kyphosis in the thoracic spine, a high C7 slope, and a high C2-7 SVA.

## Background

Diffuse idiopathic skeletal hyperostosis (DISH) is a non-inflammatory osteophytic disease of the ligaments and entheses that can affect the entire body. The unique pathology of DISH has been recognized to increase the risk of spinal disorders in affected and adjacent regions [1–3]. When a fracture occurs in the area of a DISH lesion, the structure of the spine destabilizes, possibly leading to a spinal cord injury. A predisposition to ossification has been known to influence not only activities of daily living but also quality of life [4, 5]. Notably, the spinal ankylosis caused by DISH may lead to impaired posture [6], also called sagittal imbalance, throughout the entire spine. However, very few studies [7, 8] have evaluated spinal sagittal balance according to whether or not DISH is present. Therefore, to clarify the effect of DISH on radiologic parameters related to sagittal alignment, we retrospectively investigated the prevalence of DISH in patients with cervical myelopathy caused by cervical OPLL or cervical spondylosis and compared the sagittal alignment of the spine between those with and without DISH, using propensity score matching.

## Methods

### Patient selection

The study participants comprised 103 consecutive patients with a diagnosis of myelopathy caused by cervical OPLL or cervical myelopathy who were treated at our hospital from 2014 to 2018. Patients with a history of spine surgery or injury were excluded. The study was approved by the ethics committee of our institution (#M2017-118). Informed consent was obtained from all patients before enrollment in the study.

### Clinical data and radiologic evaluation

Information was retrospectively collected for each patient on age, sex, body mass index (BMI), presence of diabetes mellitus, and whether or not there was a diagnosis of neurologic deterioration.

**Fig. 1:**
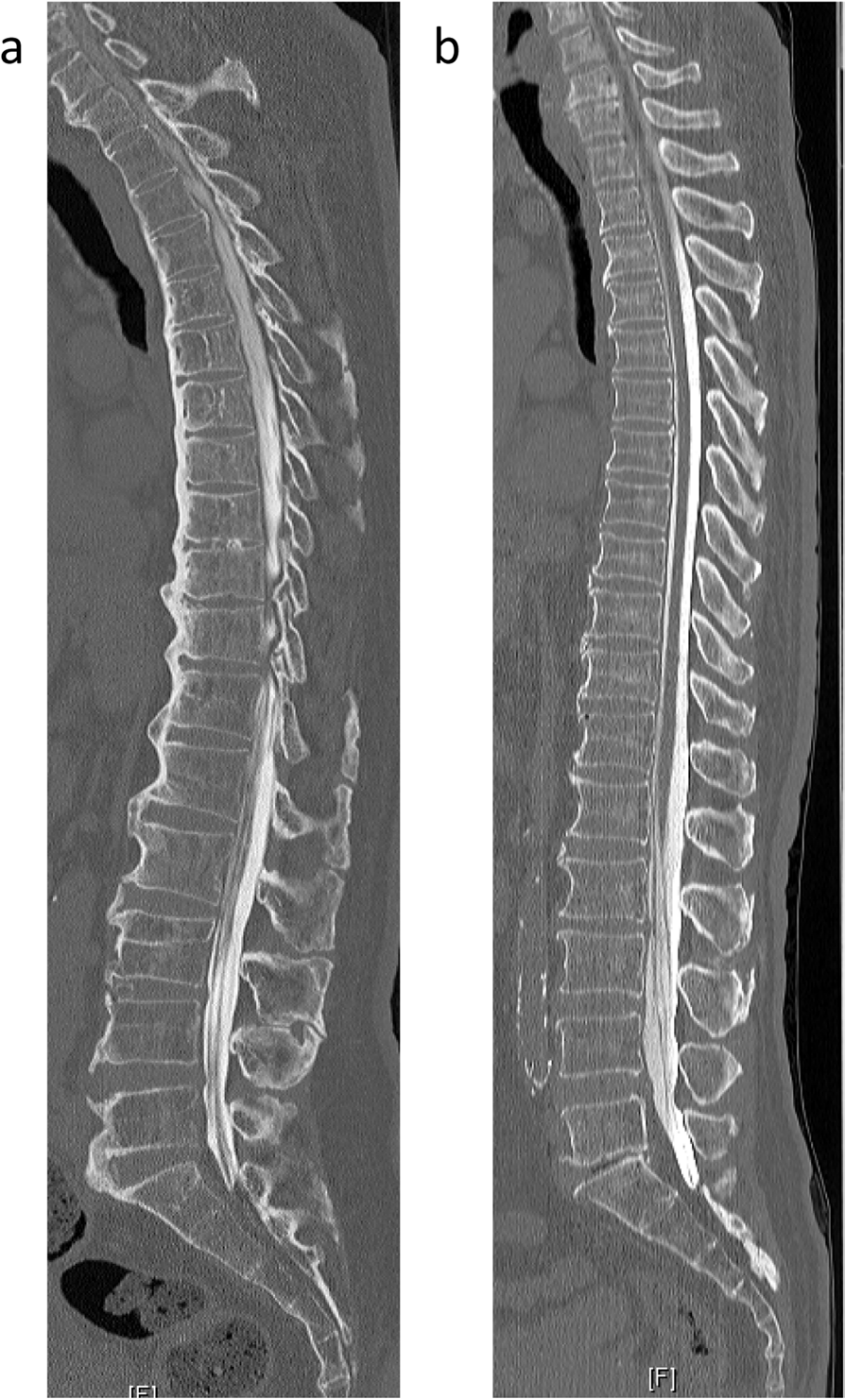
Reconstructed sagittal computed tomography images. **a** Patient with DISH. **b** Patient without DISH. DISH, diffuse idiopathic skeletal hyperostosis

Cervical sagittal alignment (C2-7 lordotic angle) was assessed by tangential lines drawn on the posterior edge of the C2 and C7 vertebral bodies on lateral radiographs acquired in a neutral position. Sagittal parameters on plain radiographs, including the C7 slope [9], C2-7 sagittal vertical axis (C-SVA) [10], kyphotic angles (w-TK) in the thoracic spine (Th1-12), upper thoracic kyphosis (u-TK) at Th1-5, thoracic kyphosis (TK) at Th5-12, and spinopelvic parameters [11], such as the SVA, sacral slope (SS), pelvic tilt (PT), pelvic incidence (PI), lumbar lordosis (LL), and PI-LL, were measured. Although DISH criteria was originally established based on plain X-ray by Resnick and Niwayama [12], DISH was defined as an ossified lesion that was found on whole spine computed tomography (CT) scans to completely bridge at least four contiguous adjacent vertebral bodies anywhere in the thoracic spine in this study (Fig. 1).

### Statistical analysis

Patients were divided into a DISH (+) group and a DISH (−) group according to the presence or absence of DISH. The two groups were compared using the chi-squared test, Mann Whitney *U* test, and Wilcoxon rank sum test as appropriate. Propensity score matching was performed for age, sex, BMI, and whether or not diabetes mellitus was present. Propensity scores were generated to compare the sagittal alignment of patients with DISH and those without DISH in a multivariable logistic regression model. All data are shown as the mean ± standard deviation. All statistical analyses were performed using SPSS for Windows version 22.0 (IBM Corp., Armonk, NY). A *p*-value < 0.05 was considered statistically significant.

## Results

### Patient demographics

There were 28 patients in the DISH (+) group and 75 in the DISH (−) group. The mean age was 64.5 years in the DISH (+) group and 62.5 years in the DISH (−) group. There was a greater proportion of men in the DISH (+) group (82.1% vs 65.3%). The mean BMI and proportion of patients with diabetes mellitus were 26.8 and 46.4%, respectively, in the DISH (+) group and 25.4 and 29.3% in the DISH (−) group. Patients in the DISH (+) group were relatively older and had a higher prevalence of diabetes compared with those in the DISH (−) group; however, the differences were not statistically significant. A significantly higher proportion of patients in the DISH (+) group had OPLL (78.5% vs 49.3%; Table 1).

**Table 1:** Demographic and clinical characteristics of the study population before propensity score matching

	DISH (+) group	DISH (−) group	*P*-value
	(*n* = 28)	(*n* = 75)	
Age (years)	64.5 ± 9.6	62.5 ± 10.5	0.44
Male sex, n (%)	23 (82.1)	49 (65.3)	0.10
Body mass index	26.8 ± 5.2	25.4 ± 3.5	0.27
Diabetes, n (%)	13 (46.4)	22 (29.3)	0.13
OPLL, n (%)	22 (78.5)**	37 (49.3)	0.008
Spondylosis, n (%)	6 (21.5)	38 (50.7)	

Data are shown as the mean ± standard deviation or as the number (percentage). **P < 0.01. *DISH* diffuse idiopathic skeletal hyperostosis, *OPLL* ossification of posterior longitudinal ligament

### Between-group comparisons after propensity score matching

All patients were included in the propensity score calculation because of differences in the baseline demographic data between the study groups. The c-statistic was 0.71 (95% confidence interval 0.672–0.821). One-to-one matching resulted in 26 pairs of patients in the DISH (+) and DISH (−) groups (Table 2).

**Table 2:** Demographic and clinical characteristics of the study population after propensity score matching

	DISH (+)	DISH (−)	*P*-value
	(*n* = 26)	(*n* = 26)	
Age (years)	64.2 ± 9.6	62.7 ± 8.8	0.55
Male sex, n (%)	21 (81)	15 (58)	0.07
Body mass index	26.3 ± 4.4	26.6 ± 3.5	0.78
Diabetes, n (%)	11 (42)	12 (46)	0.78
OPLL, n (%)	20 (77)	25 (96.2)	0.10
Spondylosis, n (%)	6 (23)	4 (3.8)	

Data are shown as the mean ± standard deviation or as the number (percentage). *DISH* diffuse idiopathic skeletal hyperostosis, *OPLL* ossification of posterior longitudinal ligament

There was no significant between-group difference in age, sex, BMI or prevalence of diabetes mellitus. The prevalence of OPLL was relatively higher in the DISH (+) group than in the DISH (−) group but the difference was not statistically significant. Sagittal parameters were then compared between the two study groups using spinal radiographs (Table 3). The C7 slope, C-SVA, w-TK, u-TK, and TK values were significantly higher in the DISH (+) group than in the DISH (−) group. There was no significant between-group difference in the C2–C7 angle, SVA, SS, PT, PI, LL, or PI-LL values.

**Table 3:** Spinal sagittal alignment according to whether or not DISH was present

	DISH (+) group	DISH (−) group	*P*-value
	(*n* = 26)	(*n* = 26)	
C2-7 angle (°)	7.2 ± 11.1	4.6 ± 10.7	0.28
C7 slope (°)	28.6 ± 11.7	19.9 ± 8.9	**0.004**
C-SVA, mm	28.6 ± 12.2	20.4 ± 10.8	**0.009**
w-TK (°)	42.7 ± 9.5	31.9 ± 8.5	**< 0.001**
u-TK (°) at T1-T5	14.1 ± 7.0	9.8 ± 5.9	**0.03**
TK (°) at T5-T12	28.6 ± 7.8	22.2 ± 8.6	**0.01**
SVA, mm	23.5 ± 37.0	26.1 ± 30.8	0.49
SS (°)	31.0 ± 6.4	32.0 ± 8.0	0.49
PT (°)	14.2 ± 7.2	13.8 ± 7.9	0.83
PI (°)	45.1 ± 7.5	45.7 ± 7.9	0.6
LL (°)	44.2 ± 10.7	40.7 ± 12.4	0.31
PI-LL (°)	12.1 ± 7.3	11.8 ± 7.4	0.2

Data are shown as the mean ± standard deviation. Bold indicates statistical significance at *P* < 0.05. *DISH* diffuse idiopathic skeletal hyperostosis, *LL* lumbar lordosis, *PI* pelvic incidence, *PT* pelvic tilt, *SS* sacral slope, *SVA* sagittal vertical axis, *TK* thoracic kyphosis at Th5-12, *u-TK* thoracic kyphosis at Th1-5, *w-TK* thoracic kyphosis at Th1-12

## Discussion

The prevalence of musculoskeletal disorders has been steadily increasing in parallel with our aging society [13, 14]. DISH is a pathologic state identified by heterotopic ossified bridging in the thoracic spine [15] and is also a systemic condition characterized by ossification of entheses throughout the body [16–18]. In a study of 504 healthy volunteers, Banno et al. [19] demonstrated on lateral radiographs that DISH had a prevalence of 14.3% in men and 4.3% in women. Similarly, Hirasawa et al. [20] reviewed 558 patients with an average age of 66.7 years who underwent CT chest to pelvis and found that the prevalence was 38.7% in men and 13.9% in women. Therefore, spine surgeons and physicians should recognize the high prevalence of DISH in elderly patients.

Few groups [7, 8] have investigated the association between sagittal alignment and the presence of DISH. Yamada et al. [7] investigated a patient population in which lumbar spine surgery was required and demonstrated that patients with DISH had significantly more thoracic kyphosis and less lumbar lordosis compared with patients without DISH. Uehara et al. [8] investigated sagittal spinal parameters and the occurrence of DISH in 411 patients from a registry that included data on a population of 5352 and showed that those with DISH in the thoracic spine had a higher sagittal vertical axis, a higher T1-slope, and more thoracic kyphosis compared with those without DISH. In the present study, we found marked differences in thoracic kyphosis and in the cervical vertical axis and C7 slope. Interestingly, we did not find a significant difference in the C2-7 lordotic angle or in any spinopelvic parameter, including SS, PT, PI. and LL, between those with DISH and without DISH. These findings indicate that patients with cervical myelopathy mostly posture cervical straight alignment with reciprocal lumbar lordosis to prevent the spinal cord compression in the canal of the cervical spine.

DISH often coexists with ossification of the spinal ligaments, including the posterior longitudinal ligament, ligamentum flavum, and interspinous and supraspinous ligaments [21–25]. Nishimura et al. [25] showed that the prevalence of DISH was as high as 48.7% in patients with cervical OPLL. They also speculated that bony bridging may develop at the thoracic level as a result of DISH and progress to the cervical and/or lumbar spine with aging. Similarly, 22 (37.3%) of the 59 patients with DISH in the present study also had cervical OPLL. Katzman et al. [26] collected data for 1500 men from the Osteoporotic Fractures in Men study and for 1267 women from the Study of Osteoporotic Fractures and assessed the prevalence of DISH and follow-up radiographs for approximately 4 years to evaluate the change in sagittal alignment in these populations. They identified DISH in 15% of men and 12% of women and found no significant difference in kyphotic change between those with and without DISH during follow-up. Interestingly, they also demonstrated that women with DISH had less kyphotic change in the thoracic spine over 15 years than those without DISH. Hirai et al. [27] prospectively collected data for 239 patients with cervical OPLL and categorized them into three grades according to the number of levels with OPLL using the cervical OP index. They found that the OP index grade was significantly correlated with extension of DISH. Similarly, Yoshii et al. also investigated a relation between the prevalence of ossification of anterior longitudinal ligament (OALL) and ossification of nuchal ligament (ONL) in cervical OPLL patient dataset, and demonstrated that the existence of ONL is significantly associated with age, male, cervical and thoracic OALL [24]. The consistent findings regarding coexistence of ossified lesions throughout the spine in patients with DISH suggest a need to consider confounding factors when assessing the association between clinical findings and radiologic findings. Although longitudinal investigation will be needed in the future, we assume that DISH may extend from the thoracic spine proximally and/or caudally with advancing age.

Patients with severe kyphosis often complain of a variety of symptoms, including severe low back pain, depression, gastroesophageal reflux caused by excessive pressure on the abdomen, and difficulty gazing straight ahead. Thoracic kyphosis has generally been considered to be a spinal deformity resulting from vertebral fractures, which are prevalent in the elderly because of osteoporosis. Katzman et al. [28] demonstrated that thoracic hyperkyphosis in the elderly is correlated with loss of muscle mass at the lower lumbar levels. It has also been shown that the range of motion at the lumbar spine is more limited in patients with DISH than in those without DISH [29]. Although the mechanisms that cause excessive kyphosis in patients with DISH are yet to be investigated, it is likely that bony bridging of the anterior longitudinal ligament gradually progresses with worsening of sagittal imbalance. Furthermore, high C-SVA associated with thoracic hyperkyphosis often influences postoperative clinical neurologic outcomes as well as cervical alignment change after cervical spine surgery. Sakai et al. reported that neurological recovery after cervical laminoplasty was poor in cervical myelopathic patients with preoperative high C-SVA [9, 30]. In addition, we previously demonstrated that graft dislodgement likely occur after anterior cervical surgery in cervical OPLL patients with high C-SVA caused by ankylosing thoracic spine [31]. Therefore, attentions to sagittal alignment in not only the thoracolumbar spine but also global spine including the cervical spine should be paid in DISH patients with spine related disorders.

DISH has been recognized to be not only a systemic skeletal abnormality affecting the entire body but also a result of metabolic syndrome. Okada et al. [32] reviewed 327 patients who attended for a routine wellness check and found that the prevalence of metabolic syndrome was significantly higher in patients with DISH than in those without DISH (28.9% vs 16.0%). Furthermore, in a study by Lantsman et al. [33] the cross-sectional area of visceral fat on abdominal CT scans was greater in patients with DISH than in healthy control subjects. Fassio et al. [34] reported that 20.8% of 96 postmenopausal women with type 2 diabetes mellitus had DISH and that the serum sclerostin level was lower in the women with DISH than in those without DISH. In the present study, although not a statistically significant finding, the prevalence of diabetes mellitus was higher (46.4%) in the DISH group. Furthermore, it has been reported that patients with DISH have an increased risk of coronary artery calcification [35, 36]. These findings suggest that adipokines might be associated with not only obesity but also ectopic bone formation in patients with diabetes mellitus, raising the possibility of a link between the onset and extent of DISH and a systemic metabolic disorder.

This study has several limitations. First, it had a cross-sectional rather than a longitudinal design, so no conclusions could be reached concerning causality. Second, it included a patient population with a specific disease; therefore, further investigations are needed in the general population to accumulate more evidence. Nevertheless, despite these limitations, we believe that our findings provide important information on the radiologic features of DISH.

## Conclusion

This study is the first to compare sagittal alignment in patients with cervical myelopathy according to whether or not they have DISH. We found that patients with DISH were more likely to have excessive kyphosis in the thoracic spine, a high C7 slope, and high C-SVA.

## Data Availability

The data and materials mentioned in this report may be made available upon reasonable request via an e-mail to the corresponding author.

## Abbreviations

BMI: Body mass index
CT: Computed tomography
DISH: Diffuse idiopathic skeletal hyperostosis
LL: Lumbar lordosis
OPLL: Ossification of the posterior longitudinal ligament
PI: Pelvic incidence
PT: Pelvic tilt
SS: Sacral slope
SVA: Sagittal vertical axis
